# The challenges of vaccine strain selection

**DOI:** 10.7554/eLife.62955

**Published:** 2020-10-13

**Authors:** Amanda C Perofsky, Martha I Nelson

**Affiliations:** Division of International Epidemiology and Population Studies, Fogarty International Center, National Institutes of HealthBethesdaUnited States

**Keywords:** influenza, evolution, prediction, phenotypes, antigenic drift, vaccines, Virus

## Abstract

New measures of influenza virus fitness could improve vaccine strain selection through more accurate forecasts of the evolution of the virus.

**Related research article** Huddleston J, Barnes JR, Rowe T, Xu X, Kondor R, Wentworth DE, Whittaker L, Ermetal B, Daniels RS, McCauley JW, Fujisaki S, Nakamura K, Kishida N, Watanabe S, Hasegawa H, Barr I, Subbarao K, Barrat-Charlaix P, Neher RA, Bedford T. 2020. Integrating genotypes and phenotypes improves long-term forecasts of seasonal influenza A/H3N2 evolution. *eLife* **9**:e60067. doi: 10.7554/eLife.60067

Scientists have known since the 1940s that influenza vaccines that perform well one year can be rendered ineffective after the influenza virus mutates. However, despite decades of investment in global surveillance, pathogen sequencing technologies and basic research (Figure 1), vaccines for seasonal influenza have the lowest and most variable performance of any vaccine licensed for use in the United States (CDC, 2016). Now, in *eLife*, John Huddleston of the Fred Hutchinson Cancer Research Center (FHCRC) and the University of Washington, Trevor Bedford of the FHCRC, and colleagues in the United States, United Kingdom, Japan, Australia and Switzerland present an open-source framework that synthesizes a decade’s worth of innovations in bioinformatics and technology to advance data-driven vaccine design (Huddleston et al., 2020).

**Figure 1.**
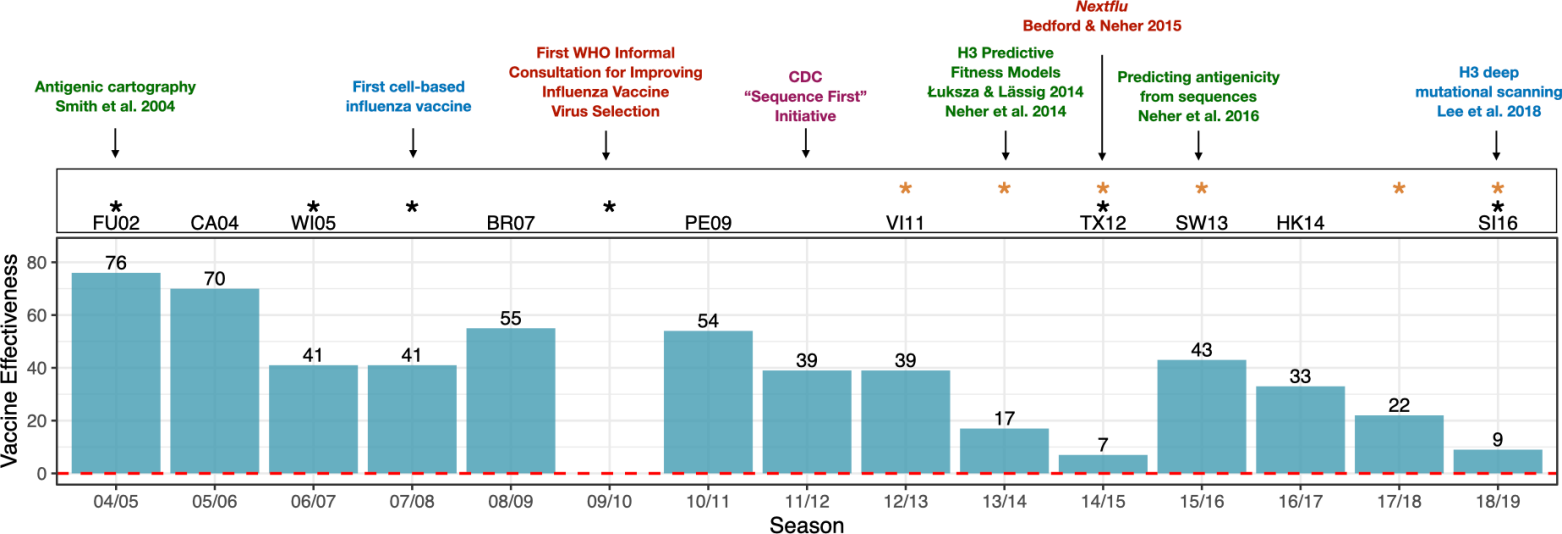
Advances in influenza research and vaccine effectiveness (for A/H3N2) from the 2004/05 flu season onwards. The effectiveness of vaccines for seasonal influenza (A/H3N2) is highly variable and has been less than 10% in some years (teal bars). The H3N2 vaccine strain is shown for seasons when it was changed from the previous season. Black stars indicate seasons where the vaccine strain mismatched circulating H3N2 viruses (https://www.cdc.gov/flu/season/past-flu-seasons.htm); yellow stars indicate seasons in which H3N2 vaccine strains acquired mutations during passage in eggs. Research advances are listed at the top of the figures and are color coded as follows: surveillance in red; experimental approaches in blue; sequencing approaches in purple; computational approaches in green. Advances in understanding the structure of hemagglutinin (Knossow et al., 1984; Wiley and Skehel, 1987) and predicting the evolution of H3 (Bush et al., 1999) occurred before the period shown in the figure. Point estimates of vaccine effectiveness are taken from the following references: Skowronski et al., 2005 (04/05); Skowronski et al., 2007 (05/06); Skowronski et al., 2009 (06/07); Belongia et al., 2011 (07/08); Skowronski et al., 2010 (08/09); Treanor et al., 2012 (10/11); Ohmit et al., 2014 (11/12); McLean et al., 2015 (12/13);; Gaglani et al., 2016 (13/14); Flannery et al., 2016 (14/15); Jackson et al., 2017 (15/16); Flannery et al., 2019 (16/17); Rolfes et al., 2019 (17/18); Flannery et al., 2020 (18/19); estimates were not available during the 2009/10 A/H1N1 pandemic.

Influenza A and influenza B viruses cause seasonal epidemics every winter. Seasonal influenza A viruses include two different subtypes, H1N1 and H3N2, where H and N (short for hemagglutinin and neuraminidase) are proteins found on the surface of the virus. The human immune system protects the body against influenza infection by producing antibodies that can recognize these proteins. However, the influenza virus mutates frequently, including at sites that affect the immune system's ability to detect the virus. This process – called 'antigenic drift' – helps the virus infect new hosts and spread in populations that previously had immunity to influenza. Indeed, antigenic drift can lead to new strains of the virus that completely displace the currently circulating strains in a matter of months.

To keep pace with antigenic drift, the composition of influenza vaccines must be updated continually. Influenza vaccines contain three or four components that protect against various strains representing the different subtypes. Scientists convene twice a year at the World Health Organization (WHO) to predict which strains will have the highest fitness and therefore dominate the next year's flu season. H3N2 viruses evolve particularly fast and unpredictably compared to other seasonal flu viruses. Because the composition of the vaccine has to be decided a year in advance to allow doses to be manufactured, H3N2 vaccine strains have failed to match naturally circulating strains in six of the past fifteen flu seasons (Figure 1).

For decades, vaccine strain selection has been primarily informed by data from 1950s-era serological assays, which provide a phenotypic measure of how immune systems exposed to recently circulating viruses would see a novel strain. However, the assays have certain disadvantages – they are labor intensive, inconsistent across labs, not publicly available, and difficult to interpret or scale up. This means that these phenotypic measures are only available for a small subset of viruses. To remedy this issue, Huddleston et al. use a phylogenetic model (which includes available serological data and sequence data as inputs) to make predictions for the thousands of strains for which serological information is not available (Bedford et al., 2014; Neher et al., 2016; Smith et al., 2004).

Huddleston et al. compare how antigenic phenotypes from serological assays perform against five newer measures of virus fitness in forecasting future H3N2 virus populations, and find that two of their models provide better forecasts than WHO vaccine strain selections. Moreover, they have now integrated their forecasts for H3N2 into nextstrain.org, an open-source platform that scientists and policymakers use to track the real-time evolution of a wide range of pathogens (Hadfield et al., 2018; Neher and Bedford, 2015). Nextstrain provides a platform to make influenza vaccine strain selection more data-driven, systematic and transparent, and to allow new forecasting methods to be integrated as they show promise.

How does one predict the fitness of an influenza virus? Most mutations are harmful for influenza viruses, except for a subset of beneficial mutations that lead to antigenic drift. For decades researchers have relied on a list of sites in the genome where seemingly beneficial mutations occur to measure antigenic drift and viral fitness (Bedford et al., 2014; Bush et al., 1999; Shih et al., 2007). However, Huddleston et al. find that serological assays (Neher et al., 2016) continue to be more useful than sequence-onlybased measures when making forecasts of future virus populations. Measures of viral fitness based on genetic sequences could not accurately predict H3N2 evolution in recent years due to the emergence of multiple co-circulating strains and the sudden decline of a dominant strain in 2019. While no method predicts the right vaccine strain every time, serology-based methods appear to outperform other approaches.

Over time, alternative approaches to measuring virus fitness will continue to be refined and may become integrated into vaccine strain selection. For example, Huddleston et al. could not include a new serological assay based on virus neutralization in their framework as data from this assay were only available over a short period of time, but it could be integrated as data accrue. Other incremental improvements could be beneficial when used in combination with serological data. For example, how fast a strain is spreading globally can be measured from branching patterns in the phylogenetic tree (Neher et al., 2014). 'Mutational load' (that is, the total number of mutations in sites unrelated to immune detection) provides a simple inverse measure of viral fitness (Luksza and Lässig, 2014), while a technique called deep mutational scanning measures whether experimentally induced mutations have beneficial or harmful effects (Lee et al., 2018), However, as with other sequence-based approaches, the fact that mutations have different effects in different genetic backgrounds may be a disadvantage.

Going forward, the COVID-19 pandemic could disrupt the ecology of flu viruses in the years ahead, and it will be interesting to observe how predictive models fare in a highly perturbed system with no historical precedent. SARS-CoV-2 viruses may also experience post-pandemic strain turnover that requires periodic updates to any COVID-19 vaccine, and it should be possible to adapt platforms built for influenza forecasting to make forecasts for SARS-CoV-2 and other pathogens.

## Note

Disclaimer: The conclusions of this study do not necessarily represent the views of the NIH or the US government.
